# Supplementary figures and images for: Prey Range and Genome Evolution of Halobacteriovorax marinus Predatory Bacteria from an Estuary
Source: mSphere. 2018 Jan 10;3(1):e00508-17. doi: 10.1128/mSphere.00508-17 (PMC5760749; doi:10.1128/mSphere.00508-17)

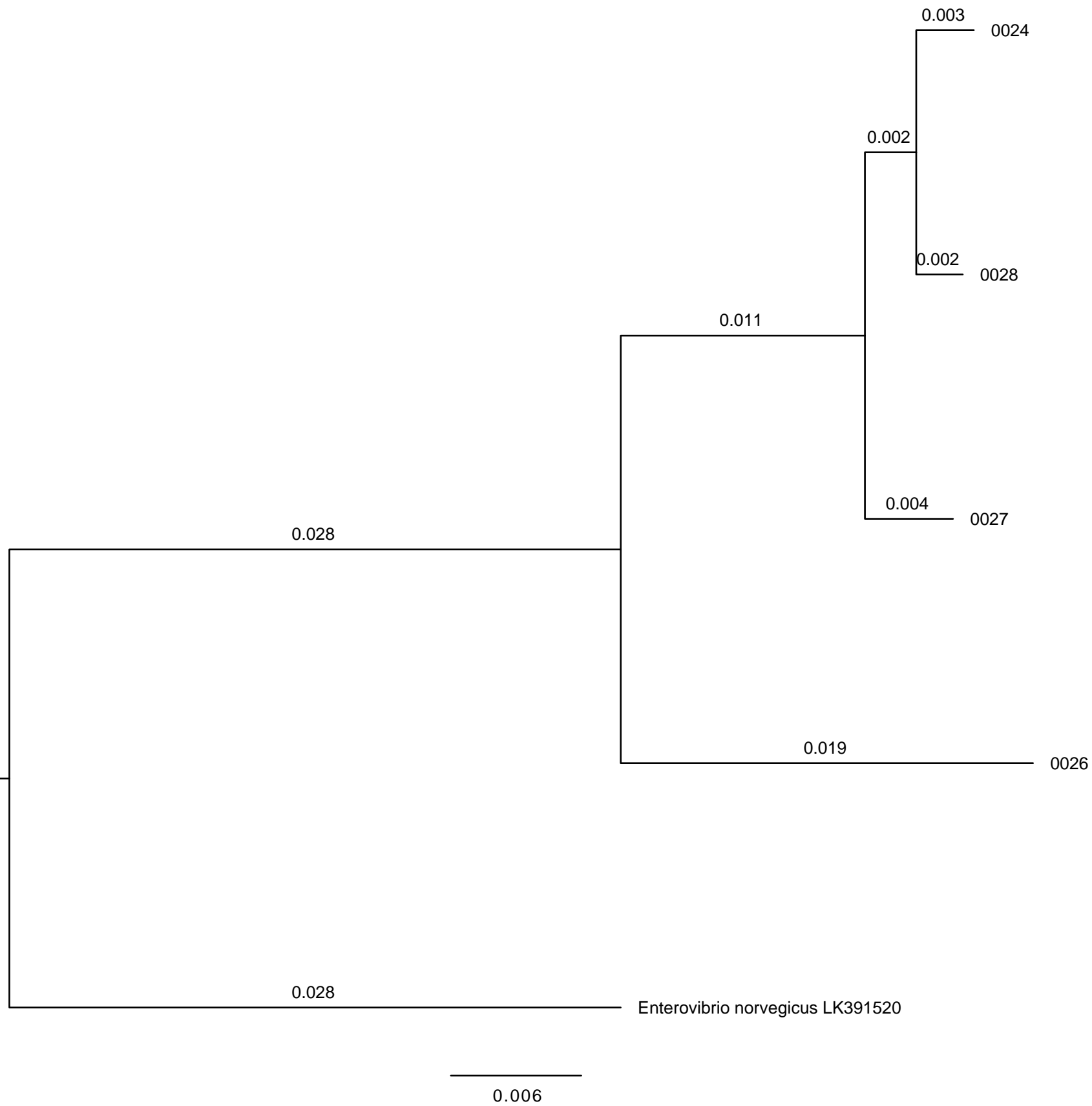

Supplement: FIG S1 [file sph001182438sf1.pdf]
